# Efficacy and safety of intravenous thrombolysis with alteplase for treating acute ischemic stroke at different time windows: A protocol for systematic review and meta-analysis

**DOI:** 10.1097/MD.0000000000023620

**Published:** 2020-12-24

**Authors:** Baogang Huang, Fang Qian, Xijun Fan, Shaoyong Guan, Yan Zheng, Junsu Yang, Fengming Xu

**Affiliations:** First People's Hospital of Qujing City, Qujing, Yunnan province, China.

**Keywords:** acute ischemic stroke, alteplase, intravenous thrombolysis, protocol, systematic review

## Abstract

**Background:**

As the priority drug for treating acute ischemic stroke (AIS), alteplase is a thrombolytic drug with strong fibrin specificity. It can obviously treat AIS with high safety. However, the validity of its time window is controversial. This study focus on the efficacy and safety of intravenous thrombolysis with alteplase for treating AIS at different time windows.

**Methods:**

Retrieval of English database (PubMed, Embase, Web of Science, the Cochrane Library) and Chinese database was conducted (China National Knowledge Infrastructure, WAN FANG, VIP, China Biology Medicine disc) by computers. From the establishment of the database to October 2020, a retrospective study and case-control study on intravenous thrombolysis at different time windows for treating AIS were conducted. Two researchers independently conducted data extraction and quality evaluation of literature on the included studies, and RevMan5.3 was used for Meta-analysis on the included literature.

**Results:**

This study aims to evaluate the efficacy and safety of intravenous thrombolysis with alteplase at different time windows for treating AIS by National Institutes of Health Stroke Scale score, modified Rankin Scale rating scale, spontaneous intracerebral hemorrhage incidence rate, All-cause mortality, and so on.

**Conclusions:**

This study will provide an evidence-based basis for the clinical efficacy of alteplase for treating AIS by thrombolytic therapy at different time windows.

**Ethics and dissemination:**

Private information from individuals will not be published. This systematic review also does not involve endangering participant rights. Ethical approval was not required. The results may be published in a peer-reviewed journal or disseminated at relevant conferences.

**OSF Registration number:**

DOI 10.17605 / OSF.IO / K7PHB

## 1 Introduction

Acute ischemic stroke (AIS) is affected by many causes (cerebral atherosclerosis, increased blood viscosity, foreign bodies, a congenital malformation of blood vessels, thickened blood vessel endothelium, formation, and rupture of arterial plaque. Arterial hemadostenosis, or even occlusion, and the formation of thrombosis caused by inflammatory and adipocyte factor), which leads to a sharp decrease of hemoperfusion of a distal cerebral artery, the insufficiency of cerebral blood supply and oxygen supply, and even lead to necrosis of brain tissue.^[1]^ Then the neurologic impairment, cognition impairment, and other symptoms appear.^[2,3]^ With a high incidence rate, high mortality rate, and high disability rate, it seriously affects residents’ physical and psychological health and burdens families and society^[4]^ the time is the life for treating cerebrovascular accident, the longer the disease time is, the worse the treatment effect.^[5]^ It is very important for early phase of AIS to achieve revascularization and restore blood flow to rescue the penumbra.^[6]^

Currently, applying recombinant tissue plasminogen activator (rt-PA) to conduct intravenous thrombolysis at a super-early stage is the most effective drug therapy to improve AIS's clinical outcome.^[7]^ As the priority drug for the treatment of AIS, Alteplase is an intravenous thrombolytic drug approved by Food and Drug Administration.^[8]^ Although its efficacy and safety have been proven clinically,^[9]^ the effective time window has always been controversial. AIS is often not found in time because of its unpredictability and suddenness. It is easy to miss the best treatment time and the formulation of therapeutic schedule. Therefore, a comprehensive understanding of the application time of thrombolytic therapy with alteplase for treating AIS.

At present, there have been many retrospective studies and case-control studies on intravenous thrombolysis by alteplase at different time windows,^[10–12]^ while the results are uneven. This study will provide an evidence-based basis for the clinical efficacy of thrombolytic therapy with alteplase for treating AIS at different time windows.

## 2 Methods

### 2.1 Protocol register

This systematic review protocol and meta-analysis have been drafted under the guidance of the preferred reporting items for systematic reviews and meta-analyses protocols.^[13]^ Moreover, it has been registered on open science framework on November 1, 2020. (Registration number: DOI 10.17605 / OSF.IO / K7PHB).

### 2.2 Ethics

Since this is a protocol with no patient recruitment and personal information collection, the ethics committee's approval is not required.

### 2.3 Eligibility criteria

#### 2.3.1 Types of studies

We will collect retrospective studies and case-control studies of intravenous thrombolysis with alteplase for treating AIS at different time windows in Chinese and English only.

#### 2.3.2 Object of study

Patients with no other anticoagulant therapies were diagnosed as AIS by brain CT or magnetic resonance imaging and other imageological examinations. There was not limitation on nationality, race, gender, age, disease time, etc.

#### 2.3.3 Intervening measure

All subjects received intravenous thrombolysis with alteplase. The dose of it was 0.9 mg/kg and the time window included ≤ 4.5 hours or >4.5 hours.

#### 2.3.4 Outcome indicator

(1) Primary outcome: ①National Institute of Health Stroke Scale score (NIHSS) (Decrease of NIHSS score (≥8 scores) 24 hours to 48 hours after the treatment or NIHSS score is 0 to 1, early progress of neurological function can be diagnosed) ②modified Rankin scale rating scales (modified Rankin Scale score of 90 days after onset, a score of 0 to 1 indicates a good prognosis, a score of 0 to 2 indicates the patient has the ability to live independently);
(2) Secondary outcomes: ①symptomatic intracranial hemorrhage risk (The incidence of spontaneous intracerebral hemorrhage as defined by the European Cooperative Acute Stroke Study II standard^[14]^) ②all-cause mortality.

### 2.4 Exclusion criteria

(1) Literature with incomplete data or little data and information cannot be analyzed;
(2) publications are abstracts and data are not available after contacting the author;
(3) animal studies, in vitro studies, case reports, reviews, etc;
(4) no relevant outcome indicators or studies with apparently wrong data;
(5) republished literature.

### 2.5 Search strategy

“Alteplase,” “rt-PA,” “acute stroke,” “AIS,” “acute cerebral infarction” in Chinese were searched in the Chinese database, including China National Knowledge Infrastructure, WAN FANG, VIP, China Biology Medicine disc. “Alteplase,” “rt-PA,” “acute stroke,” “AIS,” “AIS” in English were searched in the English database, including PubMed, EMBASE, Web of Science, and the Cochrane Library. From the database's establishment to October 2020, all domestic and foreign literature on treating AIS by alteplase at different time windows. Take PubMed as an example, the search strategy is shown in Table 1.

**Table 1:** Search strategy in PubMed database.

Number	Search terms
#1	Ateplase [title/abstract]
#2	Recombinant tissue plasminogen activator [title/abstract]
#3	Rt-PA [title/abstract]
#4	#1 or #2 or #3
#5	Stroke [MeSH]
#6	Cerebrovascular stroke [title/abstract]
#7	Stroke, cerebrovascular [title/abstract]
#8	Acute stroke [title/abstract]
#9	Stroke, acute [title/abstract]
#10	Acute cerebrovascular accident [title/abstract]
#11	Cerebrovascular accidents, acute [title/abstract]
#12	Acute ischemic stroke [title/abstract]
#13	AIS [title/abstract]
#14	#5 or #6 or #7 or #8 or #9 or #10 or #11 or #12 or #13
#15	#4 and #14

### 2.6 Filtration and extraction of data

**Figure 1:**
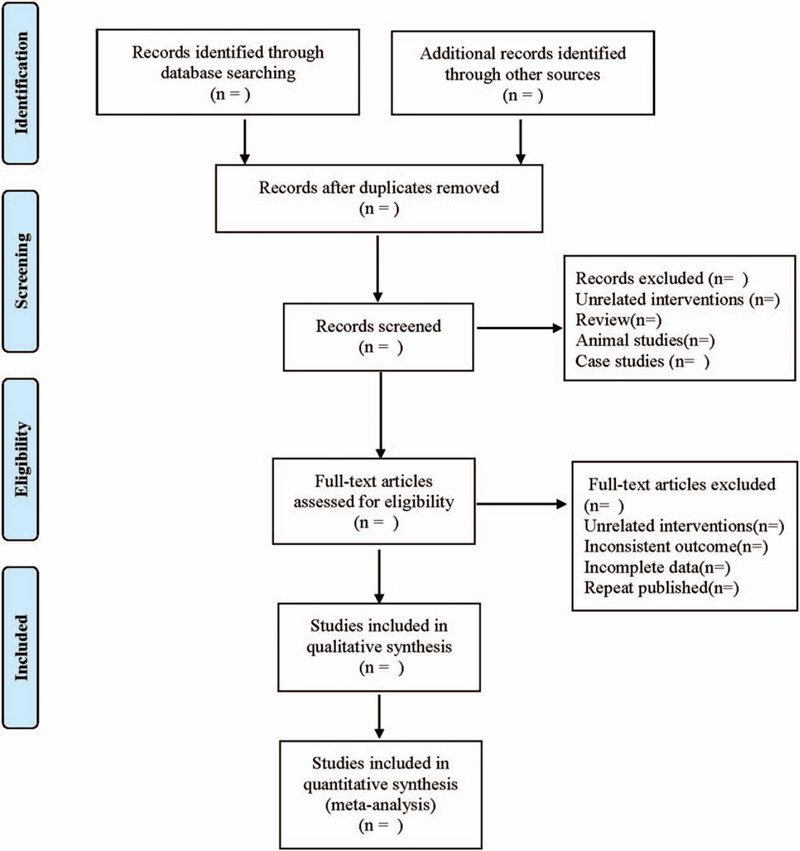
Flow diagram.

Two researchers independently extracted and checked the literature, if there were different opinions, negotiate with a third party to resolve the differences. The extraction contents included first author, year of publication, country, the start, and end time of study, number of cases, interventions, time windows, follow-up time, and outcome indicators. The selection process of the literature is shown in Figure 1.

### 2.7 Quality evaluation of literature

According to the Newcastle-Ottawa Scale, quality evaluation including 3 columns and 8 items, 9 points in total, was conducted for included studies.^[15]^ ①Selection of study population: If the determination of cases is appropriate (1 point); Representativeness of cases (1 point); Selection of comparison (1 point); Determination of comparison (1 point); ②Comparability between groups: Comparability between case and control groups was considered in the design and statistical analysis (1 point); ③Measurement of exposure factors: determination of exposure factors (1 point); the same method was used to determine exposure factors in both case and control groups (1 point); No response rates (1 point). Two researchers independently scored according to the literature's performance in the above evaluation items, and extracted and checked the literature; if there were different opinions, negotiate with a third party to resolve the differences.

### 2.8 Statistical analysis

#### 2.8.1 Data analysis and processing

RevMan 5.2 was used in Meta-analysis. Odds ratio was the effect indicator for enumeration data. The mean difference was the effect indicator for enumeration data. The point estimate and 95% confidence interval of each effect size were given. The Q check analysis was used for the heterogeneity. If *P* ≥ .10 and I^2^ ≤ 50% indicated the heterogeneity is small among studies, then the fixed effect model was used for meta-analysis. If *P < *. 1 and I^2^ > 50% indicated the heterogeneity is large among studies, then the analysis of heterogeneity sources was conducted, and the random-effects model was used for meta-analysis.

#### 2.8.2 Dealing with missing data

If there is a lack of data in the article, please contact the author by email to supplement relevant information. A descriptive analysis will be conducted if there is no way to contact authors or get relevant data. Data can be removed if necessary.

#### 2.8.3 Subgroup analysis

Subgroup analysis was conducted according to the intervention time of intravenous thrombolysis with alteplase, age of patients, and other factors.

#### 2.8.4 Sensitivity analysis

For determining the stability of outcome indicators, the sensitivity analysis was used to analyze each outcome indicator

#### 2.8.5 Assessment of reporting biases

If no fewer than 10 studies were included in an outcome measure, the funnel plots were performed to assess publication bias. Besides, Egger test and Begg test were used for the evaluation of potential publication bias.

#### 2.8.6 Grading the quality of evidence

Grading of Recommendation Assessment, Development, and Evaluation will be performed to grade the evidence.^[16]^ The content of evaluation includes bias risk, indirectness, inconsistency, inaccuracy, and publication bias. The quality of evidence will be rated as high, medium, low, or very low.

## 3 Discussion

Ischemic penumbra forms between the infarction's central necrotic area and the normal brain tissue after cerebral infarction. This part of brain tissue only has short-time viability. If blood perfusion is restored in the infarcted area at the early stage, brain tissue in the ischemic penumbra can be converted to normal tissue. If the time of ischemia is prolonged or ischemia is aggravated, irreversible necrosis will occur in the penumbra, which leads to the enlargement of the central necrotic area. The main treatment for ischemic penumbra is early thrombolysis.^[17]^ Intravenous thrombolysis for treating AIS at time windows is the recognized effective treatment around the world by rt-PA.^[18]^

In 1995, National Institute of Neurological Disorders and Stroke(NINDS) firstly proved that it is safe and effective to treat AIS by rt-PA intravenous thrombolysis after the onset within 3 hours so that 0 to 3 hours of time window can be established.^[8]^ Since then, it has been a hot spot that expanding the time window of rt-PA intravenous thrombolysis in the study so that more patients can benefit from it. The study in European Cooperative Acute Stroke Study^[19]^ and Safe Implementation of Thrombolysis in Stroke-International Stroke Thrombolysis Register^[20]^ have shown that it is also safe and effective for AIS by rt-PA intravenous thrombolysis after 3 ro 4.5 hours of onset so that the time window has been prolonged to 4.5 hours from 3 hours. However, it is lacking strong research evidence of prolonging the time window to 6 hours from 4.5 hours. Even though several studies have pointed out that intravenous thrombolysis with alteplase has a therapeutic effect for patients with acute cerebral infarction and onset time of >4.5 hours.^[21]^ Under iconography guidance, Patients who received intravenous thrombolysis for more than 4.5 hours also had a good prognosis.^[22]^ some evidence has shown that thrombolytic effect is not beneficial for patients with the onset of 4.5 to 6 hours compared with placebo group.^[23]^ It is a clinical hot spot to enlarge the time window of thrombolysis with alteplase, but the evidence is insufficient; even it is controversial that if thrombolysis is effective after the onset of 0 to 4.5 hours. This study will provide an evidence-based basis for alteplase's clinical efficacy for treating AIS by thrombolytic therapy at different time windows.

However, this system assessment also has some limitations. The limitation of alteplase's dose is 0.9 mg/kg in this study. Clinical heterogeneity can be increased for the difference of doses among parts of Asians. Due to language competence limitations, we can only search for English and Chinese literature and may ignore other languages’ studies or reports.

## Author contributions

**Data collection:** Baogang Huang, Fang Qian, and Xijun Fan.

**Data curation:** Huang baogang, Fang Qian, Xijun Fan.

**Funding acquisition:** Fengming Xu.

**Literature retrieval:** Baogang Huang, Fang Qian, and Xijun Fan.

**Resources:** Huang baogang, Fang Qian, Xijun Fan.

**Software operating:** Shaoyong Guan, Yan Zheng.

**Software:** Shaoyong Guan, Yan Zheng.

**Supervision:** Fengming Xu and Junsu Yang.

**Writing – original draft:** Baogang Huang, Fang Qian, and Xijun Fan.

**Writing – review & editing:** Shaoyong Guan, Yan Zheng, Junsu Yang, Fengming Xu.
